# Light Up the Brain: The Application of Optogenetics in Cell-Type Specific Dissection of Mouse Brain Circuits

**DOI:** 10.3389/fncir.2020.00018

**Published:** 2020-04-24

**Authors:** Candice Lee, Andreanne Lavoie, Jiashu Liu, Simon X. Chen, Bao-hua Liu

**Affiliations:** ^1^Department of Cellular and Molecular Medicine, University of Ottawa, Ottawa, ON, Canada; ^2^Department of Biology, University of Toronto Mississauga, Mississauga, ON, Canada; ^3^Department of Cell and Systems Biology, University of Toronto, Toronto, ON, Canada; ^4^Brain and Mind Research Institute, University of Ottawa, Ottawa, ON, Canada; ^5^Center for Neural Dynamics, University of Ottawa, Ottawa, ON, Canada

**Keywords:** optogenetics, neural connectivity, GABAergic neurons, neural circuit function, neural circuits and behavior

## Abstract

The exquisite intricacies of neural circuits are fundamental to an animal’s diverse and complex repertoire of sensory and motor functions. The ability to precisely map neural circuits and to selectively manipulate neural activity is critical to understanding brain function and has, therefore been a long-standing goal for neuroscientists. The recent development of optogenetic tools, combined with transgenic mouse lines, has endowed us with unprecedented spatiotemporal precision in circuit analysis. These advances greatly expand the scope of tractable experimental investigations. Here, in the first half of the review, we will present applications of optogenetics in identifying connectivity between different local neuronal cell types and of long-range projections with both *in vitro* and *in vivo* methods. We will then discuss how these tools can be used to reveal the functional roles of these cell-type specific connections in governing sensory information processing, and learning and memory in the visual cortex, somatosensory cortex, and motor cortex. Finally, we will discuss the prospect of new optogenetic tools and how their application can further advance modern neuroscience. In summary, this review serves as a primer to exemplify how optogenetics can be used in sophisticated modern circuit analyses at the levels of synapses, cells, network connectivity and behaviors.

## Introduction

In the past decades, numerous newly developed techniques have greatly assisted in dissecting connectivity and function of the brain. However, only a handful of them have influenced and advanced modern neuroscience as heavily as optogenetics. This state-of-the-art technique utilizes light-sensitive channels or pumps, known as opsins, to manipulate the activity of neurons. In addition, when it is combined with the Cre-*Lox* recombinase system, it provides a spatiotemporally precise method to reversibly turn on and off the activity of genetically defined or projection-specific groups of neurons. In this review, we will first highlight the use of optogenetics in the investigation of neural connectivity, both within and between brain regions, and then its applications in identifying the functional roles of specific neural circuit components in behavior and physiology. Finally, we will discuss some of the limitations and future directions of optogenetics. Although most of the examples in this review come from studies of sensory and motor systems, their diverse experimental designs and underlying principles are potentially useful for advancing our understanding of the structure and function of other brain circuits.

Opsins used in optogenetics were first discovered in microbes (Soliman and Trüper, 1982; Mukohata et al., 1988) and later cloned and introduced into neurons (Boyden et al., 2005). These microbial opsins can be divided into excitatory opsins and inhibitory opsins. The most commonly used excitatory opsin is channelrhodopsin (ChR2), a cation channel that opens in the presence of blue light (∼470 nm) to depolarize neurons (Nagel et al., 2003). In contrast, inhibitory opsins, such as the chloride pump halorhodopsin (eNpHR) and the proton pump Archaerhodospin (Arch), mediate hyperpolarizing currents which impede action potentials upon yellow light illumination (∼580 nm) (Zhang et al., 2007; Chow et al., 2010). In this review, we will primarily focus on the applications of commonly used opsins, rather than covering all the different variants. However, we will introduce a few recently developed opsins in order to illustrate the diverse properties of optogenetics and its unique applications.

## Connectivity

Neural circuits consist of heterogeneous cell-types receiving distinct inputs from both local and long-range sources. Dissecting the intricate connections of these neural circuits has been a long-standing challenge for neuroscientists, largely due to technical limitations in identifying and targeting specific neuronal cell-types. Although traditional methods of circuit analysis have been useful in gaining a gross understanding of macroscale and mesoscale features of brain connectivity, these techniques are limited. For example, anatomical circuit tracing with anterograde or retrograde reagents can only suggest potential innervations, without confirming the presence of functional synaptic contacts (Zeng, 2018); electron microscopy, despite its capability of identifying synapses, is labor and time intensive and cannot reveal the type or function of the synapse (Burette et al., 2015); electrical stimulation of axonal tracts, which is used to reveal functional connectivity, indiscriminately activates all fibers passing the stimulation site (Klauer et al., 1990); pairwise whole-cell recording, a gold standard for establishing connectivity, is technically challenging and time consuming and suffers from a small yield (Xue et al., 2014). Mitigating all the above issues, optogenetics, combined with the Cre-*Lox* recombinase system, provides a cell-type specific and high-throughput method for dissecting circuit connectivity.

### Local Connectivity

Neural circuits are characterized by entangled connections between various types of neurons within the network, making detailed dissection of local circuit connectivity extremely difficult. Optogenetics has simplified experimental designs for analysis of local connectivity and has greatly boosted the efficiency of data collection. For example, by selectively expressing ChR2 in a specific population of neurons, one can study the connectivity from those ChR2-expressing neurons onto other non-ChR2 expressing neurons with ease and speed (Adesnik and Scanziani, 2010; Adesnik et al., 2012). In this experimental design, optogenetic stimulation replaces the electrical stimulation in paired whole-cell clamp recordings and greatly increases the yield and the chance of detecting connectivity, since multiple presynaptic neurons can be activated simultaneously by the light-evoked current (Seeman et al., 2018) and the spatiotemporal pattern of optogenetic stimulation can be flexibly readjusted (Adesnik and Scanziani, 2010; Adesnik et al., 2012). Notably, when this optogenetic method of local circuit analysis was compared to traditional pairwise patch clamp methods, both gave rise to similar connection probabilities, validating the utility of optogenetics in the study of local circuit connectivity (Seeman et al., 2018).

**FIGURE 1:**
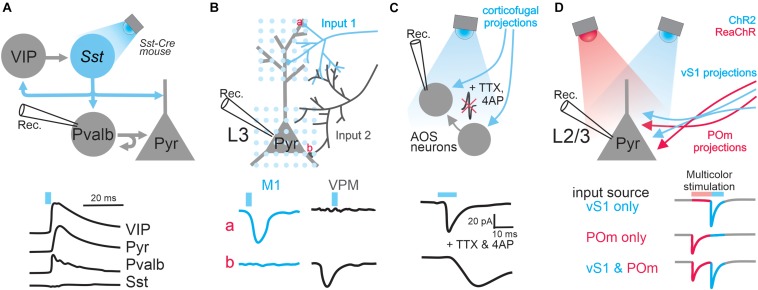
Applications of optogenetics in the analysis of circuit connectivity. **(A)** The examination of the connection from Sst positive GABAergic interneurons to excitatory pyramidal neurons and various types of GABAergic interneurons by photo-stimulating Sst neurons expressing ChR2. Top, schematic of experimental design; bottom traces, example IPSCs received by different types of cells upon Sst activation. **(B)** Subcellular ChR2-assisted circuit mapping (sCRACM) to reveal subcellular organization of two different long-range inputs impinged onto L3 pyramidal neurons. Bottom traces, example EPSCs when stimulating M1 or VPM inputs at particular domains (a, the tuft branches; b, basal dendrites). **(C)** The examination of corticofugal innervation onto brainstem AOS neurons by pharmacologically blocking action potentials. Bottom traces: example EPSCs before and after blocking action potentials. **(D)** Dual-channel mapping to resolve two different inputs received by L2/3 pyramidal neurons. ChR2 and ReaChR are expressed in axons coming from vS1 and POm, respectively. Bottom traces, the colored segments represent the EPSC components mediated by activating POm input or vS1 input. The blue or pink bars above traces represent light stimulation.

When combined with cell-type specific Cre mouse lines, optogenetics can also be used to study the connectivity of genetically-defined populations within local circuits. For instance, in the visual cortex, Pfeffer et al. (2013) examined the pattern of connectivity between three major subtypes of GABAergic inhibitory interneurons, parvalbumin (Pvalb), somatostatin (Sst), and vasoactive intestinal peptide (VIP) expressing interneurons (Figure 1A). Until recently, these different GABAergic subtypes were poorly characterized, as they are intermingled in the cerebral cortex and could not be specifically targeted with electrical or pharmacological manipulations. By expressing ChR2 in one population at a time and recording from different interneuron subtypes identified by single-cell reverse-transcription PCR, the authors were able to elucidate microcircuit motifs. They found that Pvalb interneurons preferentially inhibit pyramidal neurons and other Pvalb interneurons; Sst interneurons preferentially inhibit pyramidal neurons and all other interneuron types except themselves; and VIP interneurons preferentially inhibit Sst interneurons (Pfeffer et al., 2013). The sample sizes required to deduce connection probabilities and circuit motifs are difficult to achieve using paired recordings. But with a high-throughput optogenetic design, as the above experiment, cell-type specific connectivity analysis becomes surmountable.

Local connectivity can also be investigated between layers of the cortex. For instance, Bortone et al. (2014) used optogenetics to investigate how layer 6 (L6) excitatory neurons can regulate the strength of cortical responses throughout cortical depth. With the help of the L6 specific Cre mouse line, neurotensin receptor 1 (*NTSR1*)-Cre, they optogenetically stimulated L6 neurons and identified the recruitment of unique L6 fast-spiking interneurons with massive translaminar axons whose activation suppresses neurons across laminar layers.

### Projection-Specific Connectivity

Prior to the development of optogenetics, it was practically impossible to activate only a specific group of axonal projections. Electrical stimulation of axon tracts indiscriminately activates all fibers that pass through the stimulated area, including axons originating from different brain structures and ones projecting to different areas (Schwarz et al., 2015). Therefore, electrical stimulation will activate several pathways in parallel, which complicates data interpretation. These off-target effects can now be mitigated through the use of optogenetics. Optogenetics enables specificity at several levels: the injection location of the opsin-expressing virus provides a degree of spatial specificity for presynaptic source; the use of the Cre-*lox* system enables cell-type specificity; and the range of the light illumination provides a final level of specificity. For example, this method was successfully applied to explore the differential connectivity of thalamocortical and corticothalamic pathways that are entwined with each other (Cruikshank et al., 2010). This approach was also applied to dissect specific basolateral amygdala projections to the central nucleus of the amygdala (Tye et al., 2011).

The power of optogenetic-based projection analysis is exemplified by a study that dissected the laminar organization of long-range callosal projections linking the barrel cortices of the two hemispheres in slice preparation (Petreanu et al., 2007). Layer 2/3 of the barrel cortex receives input from several structures such as the thalamus, other cortical areas including the contralateral barrel cortex, and local circuits (Lubke and Feldmeyer, 2007). Since the axons of these inputs are intermingled in the barrel cortex, it is impossible to electrically stimulate only axons coming from the contralateral barrel cortex, namely the callosal axons, in order to investigate the laminar organization of their innervations. Therefore to address this question, Petreanu et al. (2007) unilaterally expressed ChR2 in layer 2/3 of the barrel cortex contralateral to the recording sites (Figure 1B). Because ChR2 was expressed throughout the neurons, including their axons projecting to the recording site of the barrel cortex, blue light illumination over the recording sites activated ChR2-expressing axons directly and thus stimulated only callosal input. Using this so-called ChR2-assisted circuit mapping (CRACM), the authors (Petreanu et al., 2007) systematically examined the strength of long-range callosal innervation received by neurons in individual layers of the barrel cortex and found that laminar specificity of this long-range cortical innervation is identical to local innervation (Petreanu et al., 2007). This study demonstrated that the CRACM method can reliably drive projection-specific inputs without the need to preserve their tracts in slices. However, one should be cautious of some limitations of this technique: its validation requires knowledge of the anatomy and cell types of the circuits under investigation; and severed axons in slice preparation have a limited supply of synaptic vesicles, which can be quickly depleted if one uses prolonged or particularly strong stimulation (Hass and Glickfeld, 2016).

Optogenetics can also be used to locate the synaptic innervation of long-range projections tagged by the expression of ChR2. In a subsequent study, Petreanu et al. (2009) slightly modified their CRACM protocol (Figure 1B): a blue laser beam was restricted to a small spot and raster scanned the area containing the dendritic tree of a cortical pyramidal neuron; direct activation of presynaptic terminals was achieved by pharmacologically blocking the propagation of optogenetically evoked action potentials. By recording from a postsynaptic neuron while systematically photostimulating its inputs at different locations, one can generate a 2D map of the long-range connections, a method named subcellular ChR2-assisted circuit mapping (sCRACM). With this elegant design, Petreanu et al. examined the spatial distribution of synaptic inputs onto the dendritic arborization of layer 3 and layer 5 pyramidal neurons in the barrel cortex (Petreanu et al., 2009). They found that different inputs target different apical or basal domains of those neurons.

### Establishing Polysynaptic vs. Monosynaptic Connections

Optogenetic stimulation of long-range projections can induce responses in recorded neurons via direct synaptic contacts (monosynaptic input) and/or indirectly via recurrent connections from other neurons in the network (polysynaptic input). To establish monosynaptic connectivity, one can pair ChR2 assisted optogenetic stimulation with pharmacology. By blocking voltage-gated sodium channels with tetrodotoxin (TTX) and potassium channels with 4-Aminopyridine (4AP), action potentials and thereby polysynaptic inputs will be prevented. Consequently, any remaining light-evoked response in recorded neurons must come directly from the activation of axon terminals that express ChR2. For instance, the long-range connectivity between the visual cortex and the brainstem accessory optic nuclei (AOS) was investigated by Liu et al.(2016; Figure 1C). The authors expressed ChR2 in the visual cortex and then photostimulated terminals of corticofugal axons while performing whole-cell recordings from AOS neurons. By suppressing action potentials with two different cocktails of drugs, they definitively confirmed the presence of monosynaptic connections from the visual cortex to the AOS. Without optogenetics, this experiment would not be possible, as the corticofugal axons from the visual cortex to AOS do not form a single nerve bundle, which is required for effective electrical stimulation, but instead intermingle with axons of other types of inputs. A similar design was also used to examine monosynaptic connectivity from burst-firing neurons in the subiculum to the neurons in the entorhinal cortex (Wozny et al., 2018). ChR2 was selectively expressed in burst-firing neurons by injecting the Cre-dependent *ChR2* virus into the subiculum of *VGLUT2-ires-Cre* mice, where Cre exists only in burst-firing neurons. In the presence of TTX, the authors elucidated monosynaptic connections from the axons of those burst-firing subiculum neurons with slice electrophysiology.

### Characterization of the Synapse

Beyond the identification of connectivity, when combined with pharmacology, optogenetics can be used to probe the properties of synapses. It was thought that dopaminergic projections might co-release glutamate and dopamine (Stuber et al., 2010). However, evidence supporting this idea came from studies where electrical stimulation was used to activate dopaminergic neurons, which could activate glutamatergic neurons in the neighborhood (Hnasko et al., 2010). The non-specific activation makes it difficult to discern whether the release of glutamate and dopamine indeed occur from the same terminal or two different termimals (Gu, 2010). To solve this issue, Tritsch et al. (2012) expressed ChR2, using the *Slc6a3-IRES-Cre* mouse line, in dopaminergic neurons to specifically activate dopaminergic neurons in the substantia nigra pars compacta (SNc) and pharmacologically isolated currents mediated by different neurostransmitters. Surprisingly, when they activated these neurons in the SNc and recorded from neurons in the dorsal striatum, they found both glutamatergic excitatory post-synaptic current and GABAergic inhibitory post-synaptic current, as well as amperometric dopamine transients. By combining cell-type specific optogenetics with pharmacology, they were able to definitively identify co-release of dopamine, glutamate and GABA (Tritsch et al., 2012). A similar method was also used to identify the co-release of both glutamate and dopamine by dopaminergic axonal terminals coming from the ventral tagmental area (VTA) to the prefrontal cortex (Perez-Lopez et al., 2018).

The ability to selectively activate specific projections using optogenetics also enables the characterization of synapses of local versus long-range neuronal populations. The VTA receives long-range inhibitory input from GABAergic neurons in the rostromedial tegmental area. Using ChR2, Polter et al. (2018) activated this inhitibitory input and observed that glycine receptor blocker, strychnine, significantly reduced the amplitude of inhibitory postsynaptic currents (IPSCs) in VTA neurons. They then added bicuculline, a GABA_A_ receptor blocker, along with strychnine and all residual IPSCs were abolished, suggesting this inhibitory projection co-releases GABA and glycine. They also performed the same recording with ChR2 expressed in VTA GABAergic interneurons to stimulate local inhibition, but did not find co-release of glycine (Polter et al., 2018). They proceeded to perform further characterization of these two inhibitory inputs received by VTA neurons. For example, they utilized light stimulation to compare paired-pulse ratios of those two types of inhibitory synapses, demonstrating how optogenetics can be used to characterize synaptic properties of distinct neuronal populations.

### Dual-Channel Mapping

A single neuron might receive multiple inputs coming from different regions, representing multiplexed streams of information transmission (Petreanu et al., 2009; Oh et al., 2014). Understanding this input convergence is a fundamental but difficult task since it requires the technical capability of individually manipulating different types of inputs. This independent control of different inputs is impossible with electrical stimulation when the axons of those inputs are intermingled. This technical challenge was solved through the use of dual-color optogenetics when Hooks et al. studied the convergence of two different intermingled inputs in the primary motor cortex (Hooks et al., 2015; Figure 1D). In order to achieve this, they chose two opsin variants that prefer different wavelengths of light: blue light sensitive ChR2 was used to excite axons originating from the barrel cortex and ReaChR, an opsin activated by orange light (Lin et al., 2013), to perturb input from the posterior medial thalamic nucleus. Despite the distinct optimal excitation wavelengths of the two opsins, blue light can in fact excite ReaChR as well as ChR2. Therefore, when illuminated by blue light, both pathways will be stimulated, complicating data interpretation. To solve this complication, the authors created a clever protocol that reversibly inactivates ReaChR prior to activating ChR2 (Hooks et al., 2015), allowing complete separation of the two inputs. Indeed, with whole-cell recording they found that the input from the somatosensory cortex and that from the thalamus converge on the same layer 2/3 neurons in the motor cortex. Since this optogenetic dual-channel stimulation does not require the spatial segregation of axons of different inputs – a condition demanded by electrical stimulation – this mapping method can in principle be generalized on any convergent circuit system and is especially indispensable in the case where axons of distinct origins intermingle.

*In vitro* slice electrophysiology is a gold standard to determine connectivity between pairs of neurons. Although care is taken to preserve the integrity of the circuit during slice preparation and to maintain similar physiological conditions to live animals during recording, some damage and cell death is inevitable, and impedes faithful quantification of neuronal connectivity. To address this issue, efforts had been made to examine connectivity in a physiologically pristine environment with the help of optogenetics. For example, Pala and Petersen (2015) performed *in vivo* whole-cell recordings from GABAergic interneurons in L2/3 of the barrel cortex to examine the connection from excitatory pyramidal neurons. To precisely measure unitary excitatory postsynaptic potentials (uEPSPs) in GABAergic interneurons, these researchers introduced plasmid DNA encoding ChR2 into a single L2/3 pyramidal cell with electroporation and elicited one action potential per stimulus with very brief light pulses. In particular, they compared the uEPSPs response between Pvalb and Sst inhibitory neurons, finding that each inhibitory population differed in the probability, time course, strength, reliability, and short-term synaptic plasticity of their response to excitatory stimulation. This finding largely agrees with previous *in vitro* results (Pala and Petersen, 2015).

## Functional Dissection

Thus far, we have discussed how optogenetic strategies can aid the dissection of circuit connectivity. Beyond characterizing connectivity, optogenetics is also an extremely powerful tool when investigating the functional roles of specific neural circuits in animal behavior and physiology. *In vivo* investigations present a unique set of demands and limitations. For many years, researchers have relied on extracellular recordings to investigate neural activity *in vivo*. Although this method allows monitoring activity from large populations of neurons, the type of neuron recorded cannot be identified in most cases, limiting its applications in the study of cell-type specific functions. Optogenetic tagging provides a feasible solution to this problem. For example, Lima et al. (2009) restricted the expression of ChR2 to Pvalb interneurons in the auditory cortex and inserted a recording electrode into this cortical area. When they illuminated this area with brief pulses of blue light, short latency spikes that are precisely synchronized with the light pulse were reliably elicited in a population of neurons, distinguishing them as Pvalb interneurons expressing ChR2. These authors also used this approach to identify auditory cortical neurons projecting to the contralateral hemisphere, namely callosal projection neurons, for which a retrograde herpes simplex virus-1 (HSV-1) encoding ChR2 injected to the auditory cortex in one hemisphere was used to tag callosal projection neurons in the other hemisphere (Lima et al., 2009). These two examples demonstrate the utility of this optogenetic tagging technique for both cell-type and projection-type specific functional circuit analyses.

In addition to identifying neuron type *in vivo*, optogenetics can also be used to manipulate neuronal activity during behavior. The canonical experiment to define a neural correlate of a behavior is to activate or silence the putative correlate *in vivo* and assess whether and how the behavior is altered. For decades, the field had relied on electrical stimulation/lesions and pharmacological activation/silencing, however, these techniques of circuit manipulation have intrinsic problems. They offer little to no temporal precision, nor cell-type specificity; furthermore, lesions are permanent and can result in unpredicted plasticity and compensatory mechanisms that confound results (Whishaw, 2000; Murphy and Corbett, 2009). The advent of optogenetics enables immediately reversible manipulation and allows trial-by-trial and within-animal comparisons in a single session. The remainder of the review will examine how the use of optogenetics has enabled cell-type and projection-specific circuit dissection of visual, somatosensory and motor function *in vivo*.

### Visual Cortex

Since Hubel and Weisel first discovered the fundamentals of visual processing in the primary visual cortex (Hubel and Wiesel, 1959, 1963, 1968), numerous studies have extensively examined and characterized visual processing in different model systems. These studies revealed that stimulus features such as orientation and direction are encoded in the mammalian visual cortex through tuned neural responses at both the level of single neurons and cortical columns. However, due to the prevalence of pyramidal neurons over GABAergic inhibitory neurons in the cortex, the “blind” electrophysiological recording methods used in these studies primarily characterized pyramidal neurons and could not clearly elucidate how other cell-types might contribute to this neural code.

**FIGURE 2:**
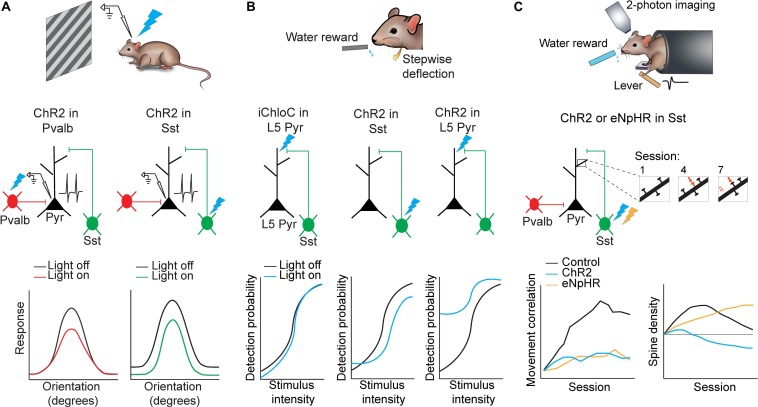
Applications of optogenetics in the analysis of circuit functions. **(A)** Mice were presented with oriented drifting gratings while *in vivo* cell-attached recordings of putative pyramidal neurons in the visual cortex were performed. Either Pvalb or Sst interneurons were photoactivated using ChR2. Pvalb interneuron photoactivation (left) resulted in divisive inhibition of pyramidal neurons where suppression was stronger at orientations where pyramidal neuron responses were also stronger. Sst interneuron photoactivation (right) resulted in subtractive inhibition where suppression was uniform along all orientations. Adapted from Wilson et al. (2012). **(B)** Mice were trained to lick for a water reward in response to whisker stimulation of varying intensities. Perceptual detection as a function of stimulus intensity formed a sigmoid curve. When L5 pyramidal neuron apical dendrites were photoinhibited (iChloC) or when Sst interneurons were photoactivated (ChR2), the curve shifted to lower detection probabilities. When L5 pyramidal neuron apical dendrites were photoactivated (ChR2), the curve shifted to higher detection probabilities. Adapted from Takahashi et al. (2016). **(C)** Mice were trained to press a lever following an auditory cue to obtain a water reward. Two-photon imaging was performed in M1 throughout learning to track dendritic spine dynamics in pyramidal neurons. Control animals developed a stereotyped lever-press movement with learning but this was impaired if Sst interneurons were photoactivated (ChR2) or photoinhibited (eNpHR) (bottom left). Furthermore Sst interneuron photoinhibition resulted in increased stabilization of dendritic spines while photoactivation resulted in increased elimination. (bottom right). Adapted from Chen et al. (2015).

A long-standing question in the field has been to understand how orientation selectivity emerges in the visual cortex. For many years it had been speculated that inhibition from GABAergic interneurons may shape tuning of pyramidal neurons (Sillito et al., 1980; Sato et al., 1996; Ringach et al., 2003; Liu et al., 2011), although findings have been inconclusive until recently since this hypothesis could not be directly examined before the invention of optogenetics and inhibitory neuron-specific mouse lines. Using high-speed calcium imaging or *in vivo* cell-attached recordings paired with subtype-specific optogenetic activation, Wilson et al. (2012) demonstrated that different subtypes of GABAergic interneurons indeed have distinct roles in shaping pyramidal neuron responses to visual stimuli. The authors expressed ChR2 in either Pvalb or Sst interneurons in the primary visual cortex in order to activate each subtype independently while showing the mouse oriented drifting gratings (Figure 2A). They also used simultaneous calcium imaging or cell-attached recordings to characterize the orientation tuning of pyramidal neurons. By comparing pyramidal neuron tuning curves with and without optogenetic activation of Sst or Pvalb interneurons, they found that Pvalb and Sst populations exert distinct computational control on the responses of pyramidal neurons. Sst interneuron activation resulted in uniform subtractive inhibition in pyramidal neurons across all directions of drifting gratings. Pvalb interneuron activation, on the other hand, led to divisive inhibition in pyramidal cells, where the effects of inhibition were strongest when the grating was at the neuron’s preferred orientation. In this way, Sst interneurons sharpen stimulus selectivity in the visual cortex, while Pvalb interneurons modulate response gain but preserve stimulus selectivity (Wilson et al., 2012). Moreover, bidirectional manipulation of Pvalb interneurons in another study using ChR2 or Arch to activate or silence Pvalb interneurons respectively, further supports the role of Pvalb interneurons in modulating the gain of pyramidal neuron responses without strongly altering tuning properties (Atallah et al., 2012). Importantly, without this cell-type specific optogenetics, it would be impossible to target or manipulate different types of neurons.

In addition to its applications in local microcircuitry, optogenetics has also been used to investigate the role of long-range inputs in visual processing. To parse the role of specific inputs to the visual cortex, Zhang et al. (2014) used a virus to express ChR2 in the cingulate cortex and then shone blue light on the visual cortex, thus exciting only the axons projecting from the cingulate cortex to the visual cortex. Activation of this top-down projection sharpened tuning of pyramidal neurons in the visual cortex and improved behavioral performance in a visual discrimination task (Zhang et al., 2014). The authors further examined which cell-types in the visual cortex might be receiving this input from the cingulate cortex. Tracing experiments revealed that Pvalb, Sst and VIP interneurons in the visual cortex all receive long-range top-down input from the cingulate cortex. Hence, to delineate subtype-specific roles in this projection, the authors again, expressed ChR2 in the cingulate cortex, but they additionally expressed the inhibitory opsin, eNpHR, in either Pvalb, Sst or VIP interneurons in the visual cortex. By patching pyramidal neurons in the visual cortex and either (i) silencing one subtype of local inhibitory neurons, (ii) activating cingulate axons, or (iii) doing both simultaneously, the effect of cingulate cortex projections on the different inhibitory populations was dissected. By taking advantage of projection-based and cell-type based optogenetics, the researchers were able to demonstrate that cingulate cortex input activates all three types of inhibitory neurons. However, Sst and Pvalb interneuron activation inhibited a broad cortical area, while VIP interneuron activation selectively enhances responses in the center region. Therefore, VIP interneurons may have a unique role of disinhibiting the center, while Sst interneurons inhibit the surround, thus explaining a facilitatory center and a suppressive surround of top-down modulation during visual processing.

### Somatosensory Cortex

Optogenetics has also been employed in the somatosensory cortex to dissect the function of specific projections during perception of sensory stimuli. By expressing the inhibitory opsin Arch in the secondary motor cortex (M2), Manita et al. (2015) used amber light in the primary somatosensory cortex (S1) to inactivate projections from M2 to S1, while simultaneously recording from S1 with either multi-unit electrodes or patch-clamp electrode. Using this preparation, the authors compared the neural response in S1 following hindpaw stimulation with and without perturbation of M2 innervation. They found that silencing M2 projections did not alter fast, putative bottom-up responses to sensory stimuli but did lead to a significant reduction in the slower second wave of top-down activity, suggesting M2 provides significant top-down modulation on S1 activity following sensory stimuli. Furthermore, using a miniature wireless LED device that can be implanted onto a freely moving, behaving mouse, they found that silencing M2 projections to S1 impaired tactile discrimination, revealing that M2 projections to S1 contribute to this type of sensory processing (Manita et al., 2015). This study demonstrates the importance of *in vivo* optogenetic approaches, since large scale network activity in response to sensory stimuli cannot be studied *in vitro*.

Another study used optogenetics in S1 to reveal the importance of dendritic activity in layer 5 (L5) pyramidal neurons in sensory perception (Takahashi et al., 2016). The authors first trained mice to lick for a reward following whisker deflection until an 80% success rate was achieved. They then constructed a psychometric curve by varying the intensity of the whisker deflection to uncover the intensity threshold for perceptual detection (Figure 2B). A multi-pronged approach was then employed to dissect the neural correlate of sensory perception in S1. Remarkably, using three distinct optogenetic approaches, the animal’s detection probability curve was shifted bidirectionally. First, the authors used iChloC, an inhibitory chloride-conducting channelrhodopsin (Wietek et al., 2015), expressed in L5 pyramidal neurons to silence apical dendrites of L5 pyramidal neurons in behaving mice, which was achieved by calibrating light intensity *ex vivo* to target superficial layers of S1 without affecting activity at the soma of L5 pyramidal neurons located deeper in the cortex. Silencing the L5 pyramidal neuron dendrites shifted the detection probability curve to higher intensity values when compared to light-off trials, implying mice had impaired detection ability. Second, to further understand the local microcircuit, the authors expressed ChR2 in Sst interneurons, which preferentially inhibit apical dendrites of pyramidal neurons. Activating Sst interneurons also significantly shifted the detection probability to higher intensities. Finally, activation of ChR2 expressed in L5 pyramidal neurons shifted the detection probability to significantly lower detection probabilities and caused a substantial increase in false detection rates. The use of optogenetics enables a reversible approach for within animal and within session comparisons between light on and light off trials.

### Motor Cortex

The motor cortex is unique among primary cortical areas in that its primary function is to activate muscles and execute movements. Inactivation techniques such as pharmacological inactivation or lesions lack the temporal resolution needed to dissect circuit contributions to specific aspects or components of a movement, such as an arm movement versus digit movement in a reaching task. Additionally, motor cortex lesions impair movement but do not result in the complete loss of movement (Castro, 1972; Alaverdashvili and Whishaw, 2008) due to compensatory mechanisms that change motor function over time (Whishaw, 2000), further obscuring motor cortex functions under normal conditions.

The ultra-fine temporal precision of optogenetics makes it an ideal tool for addressing these open questions regarding the motor cortex. In recent years, the use of optogenetics in the motor cortex has elicited a range of results, some of which do not support previous findings in lesion studies, further demonstrating how lesion and pharmacological studies can be limiting when probing complex systems conveying multiplexed information. To test the role of the motor cortex in learned behaviors, Guo et al. (2015) used a transgenic mouse line expressing ChR2 in all GABAergic neurons and trained head-fixed mice on a forelimb pellet reaching task until the mice achieved expert level. Following training, they used optogenetic activation of GABAergic inhibitory neurons to silence the central forelimb area of the motor cortex. Remarkably, they demonstrated that silencing the motor cortex before movement blocked the initiation of reaching, while silencing the same area following movement initiation caused the mouse to pause with its limb protracted. The other limbs and any untrained movements such as grooming were unaffected. In fact, turning off the motor cortex and then releasing the inhibition was sufficient to initiate reaching movement outside of trials when there was no task-related cue or food reward present, indicating that the release of inhibition could trigger the full activation sequence for the trained reach movement. Previous lesion studies have demonstrated impairments in behaviors but failed to reveal such a robust effect (Castro, 1972; Alaverdashvili and Whishaw, 2008). This can perhaps be explained by compensatory mechanisms that can contribute to recovery of movement when the motor cortex malfunctions for long periods of time (Murphy and Corbett, 2009), demonstrating the advantage of fast and reversible optogenetics.

Furthermore, learning a new movement has been shown to be associated with the emergence of a reproducible neuronal activation pattern in the motor cortex that is specific to the learned movement (Peters et al., 2014). Subcellularly, motor learning also induces the addition and selective elimination of dendritic spines on pyramidal neurons to reorganize synaptic connections in the motor cortex (Xu et al., 2009; Peters et al., 2014; Chen et al., 2015). However, until recently, the role of GABAergic interneurons in motor learning was not clear. Using a combination of two-photon imaging and optogenetics, Chen et al. (2015) demonstrated that Pvalb and Sst interneurons have distinct roles in motor learning (Figure 2C). The authors trained mice on a cued forelimb lever press task. Mice demonstrated increased success rate and highly correlated, stereotyped press movements over the course of training, hallmarks of learning. The authors then used ChR2 or eNpHR to activate or inhibit Sst interneurons respectively during each session throughout learning, while assessing learning-induced spine reorganization in pyramidal neurons in the motor cortex. Activation of Sst interneurons during motor learning led to increased elimination of newly-formed spines, thus abolishing learning-induced spine reorganization. In contrast, inhibiting Sst interneurons led to decreased elimination of newly-formed spines and hyper-stabilized the spines, thus disrupting the reorganization process. Both optogenetic activation or inhibition of Sst interneurons impaired learning and impaired the ability to form stereotyped press movements across trials. Lastly, when Sst interneurons were manipulated during the task in mice that had already been trained, the movement was unaffected, indicating that the activity of Sst interneurons may be involved in regulating structural remodeling in pyramidal neurons only during motor learning and is, therefore, most critical for the acquisition of motor memories but not the execution of previously learned movements (Chen et al., 2015).

The contribution of long-range input to motor function has also been examined using optogenetics. To understand the role of thalamus and the anterior motor lateral area (ALM), two main sources of inputs to the primary motor cortex, in movement, Guo et al. (2017), utilized potent circuit silencing strategies that activate GABAergic inhibitory neurons innervating those two brain regions. The authors used ChR2 to optogenetically stimulate GABAergic axonal terminals coming from the thalamic reticular nucleus to remove the contribution from thalamic input or, they optogenetically activated inhibitory interneurons in ALM to examine the contribution from ALM. Along with silencing either thalamic activity or ALM activity, they also conducted simultaneous multi-unit recordings in ALM or thalamus while mice performed a lick – no lick task. The task made use of a delay period between the go cue and the movement, which allowed for the assessment of preparatory activity in these regions preceding movement. Intriguingly, they found that silencing thalamus during the delay period almost entirely abolished ALM spiking. Similarly, silencing ALM strongly reduced thalamic spiking. This suggests that ALM and thalamus are connected in a recurrent loop resulting in persistent spiking. Lastly, silencing either area significantly impaired the mice’s performance in the task when contralateral movements were required but ipsilateral movements were unaffected (Guo et al., 2017), demonstrating that this loop is critical for movement preparation. Optogenetics is essential when dissecting movements that operate on millisecond to second timescales, as this type of detailed movement deconstruction and delineation is not possible with slower approaches such as pharmacology or DREADDs.

Optogenetic activation of GABAergic inhibitory neurons in the visual cortex or in the thalamic reticular nucleus has also been used to shut down local recurrent excitation or long-range thalamocortical excitation respectively, in order to elucidate their contribution to cortical visual processing (Li et al., 2013; Lien and Scanziani, 2013; Reinhold et al., 2015).

## Optogenetics in Diverse Animal Models

This review has primarily focused on the use of optogenetics in mice because of the extensive availability of transgenic mouse lines that permit cell-type specific circuit dissection. However, it is important to note that optogenetics can also be used in other animal models. In particular, rats present a desirable animal model, because they can learn more complex tasks, and because their larger size, relative to mice, enables them to tolerate implants more easily. Recently, more and more transgenic rat lines have become available, which makes the aforementioned cell-type specific experimental designs feasible for this animal model. For example, Witten et al. (2011) used *Tyrosine hydroxylase (Th):Cre* transgenic rats to drive the expression of the Cre recombinase under the dopaminergic specific *Th* promoter. By injecting Cre-dependent *ChR2* virus in the VTA, the expression of ChR2 was restricted to dopaminergic neurons in the VTA. With this model, they investigated cell-type specific circuitry driving positive reinforcement during intracranial self-stimulation (ICSS). During a freely moving ICSS task, optogenetic stimulation of dopaminergic neurons in the VTA occurred when rats nosepoked one of the two identical ports, but not when they poked the other one. This simple optogenetic activation of dopaminergic system during behavior strongly biased rats to the port associated with optogenetic stimulation, but not the other port, thus demonstrating that activation of dopaminergic cells in the VTA is sufficient to drive ICSS.

Optogenetics has also been used in rats to investigate projection-specific contributions to motivation. For instance, Warden et al. (2012) targeted medial prefrontal cortex (mPFC) neurons that project to the dorsal raphe nucleus (DRN) by virally expressing ChR2 in mPFC neurons and photostimulating their axons in the DRN. The authors used a protocol of alternating 2 min light-on epochs followed by 2 min light-off epochs for five cycles during the forced swim test, allowing multiple within session comparisons. They observed a robust increase in kick frequency during light-on epochs relative to light-off epochs. In contrast, when they activated the mPFC-lateral habenula pathway in the same way, kick frequency during light-on epochs was robustly decreased. These projection-specific, opposing behavioral phenomena were not seen when all mPFC neurons were non-selectively photostimulated. These results demonstrate that optogenetic activation of projection-specific pathways arising from the same brain area can result in opposite behavioral effects and further corroborate the need for circuit specific perturbation in behavioral studies.

Optogenetics has also been used in higher mammals, such as Rhesus monkeys, enabling the causal study of circuits involved in complex behaviors and neurological diseases (Han et al., 2009; Jazayeri et al., 2012; Shewcraft et al., 2020). In the first study that validated the use of optogenetics in non-human primates, Han et al. used lentivirus to express ChR2 in the frontal cortex of macaques, and established that viral expression of opsins is safe and can support long-term experiments (Han et al., 2009). Even though there is a lack of genetically modified Cre-expressing monkey lines, cell-type specific promoters can be used to target unique neuronal populations. For instance, Stauffer et al. (2016) used virus with a dopaminergic cell-specific *Th* promoter to express Cre recombinase alongside a cre-dependent *ChR2* virus, thus restricting ChR2 expression to dopaminergic neurons in the midbrain. They then demonstrated that the activation of this dopaminergic population promotes reward-related learning at both cellular and behavioral levels. Similarly, the Purkinje cell specific promotor *L7/Pcp2* has been used to selectively drive ChR2 expression in Purkinje cells in the cerebellum occulomotor vermis (El-Shamayleh et al., 2017), and the *CaMKII* promotor has been used to express ChR2 in pyramidal neurons in the frontal cortex (Han et al., 2009). In addition to cell-type specificity, projection-specific optogenetics has also been achieved in Macaque monkeys. Galvan et al. expressed ChR2 in the primary motor cortex of macaque monkeys and optogenetically activated the terminals of the corticothalamic projection from the motor cortex, revealing that this projection pathway plays a modulatory role in the thalamus (Galvan et al., 2016). These studies in non-human primates demonstrate the wider applicability of optogenetics for circuit and behavior analysis with both projection (Inoue et al., 2015; Galvan et al., 2016) and cell-type specificity (Han et al., 2009; Jazayeri et al., 2012; Klein et al., 2016; Nakamichi et al., 2019).

## Future Directions

### New Variants of Opsins

Over the past decade, several newly developed variants of opsins have enabled the use of optogenetics in investigations that were not technically possible with earlier variants. Although this review is not intended to provide a comprehensive overview of different opsins, this section will highlight some experimental designs that leveraged unique properties of engineered opsin variants.

When first introduced into neurons, ChR2 was demonstrated to drive spike trains ranging from 5 to 30 Hz (Boyden et al., 2005). However many neurons are capable of firing far beyond 30 Hz (O’Connor et al., 2010). High-speed opsins are indispensable to investigate fast spiking neuron types such as Pvalb interneurons (Chrobak and Buzsaki, 1998; Hajos et al., 2004); hence, oChIEF was developed in 2009 (Lin et al., 2009), ChETA developed in 2010 (Gunaydin et al., 2010), and Chronos developed in 2014 (Klapoetke et al., 2014). These ChR2 variants can reliably evoke ultra fast spiking in neurons, up to 100 Hz.

One advantage of these ultra fast opsins was demonstrated when studying the importance of long-term potentiation (LTP) for memory formation (Bliss and Lomo, 1973). Although this hypothesis had existed for many years, it was difficult to test it directly *in vivo*, because of the lack of tools to induce LTP in live animals. A classical LTP protocol for slice preperation involves high-frequency tetanic electrical stimulation (a train of brief pulses at 50–100 Hz) of the tract of presynaptic axons, which is not feasible for *in vivo* experiments. However, the ultra fast opsins which support reliable optogentic stimulation up to 100 Hz provide an opportunity. Nabavi et al. (2014) used AAV to express oChIEF in the medial geniculate nucleus and the auditory cortex of rats and implanted a cannula in the lateral amygdala to deliver light to the oChIEF expressing axon terminals originating from the medial geniculate nucleus and auditory cortex. The authors then delivered five trains of brief light pulses at 100 Hz and used *in vivo* field recordings to confirm the induction of LTP. When this optical stimulus used to induce LTP, was paired with a foot shock in a cued fear conditioning paradigm, the rats performed the conditioned fear response. In contrast, when the optical stimulus and the shock were unpaired, the rats did not perform the conditioned fear response, suggesting they had not formed the cued-fear memory. In this experiment, the reliable high frequency optogenetic stimulation of axon terminals depends on several desirable properties of oChIEF, such as fast opening and closing rates, high light sensitivity and the low channel inactivation (Lin, 2011; Hass and Glickfeld, 2016).

In addition to ChR2 variants with faster kinetics, step function opsin (SFO) (Berndt et al., 2009) and stabilized step function opsin (SSFO) (Yizhar et al., 2011) have been engineered to produce very slow kinetics, which confer a unique set of experimental advantages. These opsins have mutations at the C128 position in ChR2 that substantially extend the period of activation. They are also capable of bistable switching, in which they can be activated with blue light to produce prolonged depolarization that can be rapidly terminated with amber light. While SFO can support stable depolarization for minutes (Berndt et al., 2009), SSFO mediated depolarization is stable over the 30 min time scale (Yizhar et al., 2011). This unique property makes SSFO useful for *in vivo* investigations since complex animal behaviors typically occur on the timescale of minutes. In addition, SSFO can also be useful when optogenetics is paired with two-photon calcium imaging, since it solves the issue of visible light from optogentic stimulation interferring with GCaMP based calcium imaging. For example, Makino and Komiyama (2015) used a virus carrying a Cre-dependent *SSFO* gene to conditionally express SSFO in Sst inhibitory neurons of *Sst-Cre* mice, and expressed GCaMP6f in pyramidal neurons. With this design, the researchers optogenetically turned Sst interneuron activity on (using blue light) and off (using amber light), while performing calcium imaging of mostly pyramidal neurons. The use of SSFO in this study enabled cell-type specific optogenetic manipulation throughout the entire course of a complex behavioral task with simultaneous calcium imaging. This study also exemplifies the flexibility of optogenetics, enabling within animal and within session controls.

The level of control achieved through optogenetics can be extended even further using the opto-XR family of opsins. This family of opsins was engineered by replacing the intracellular loops of rhodopsin with those from specific G-protein coupled receptors, allowing photoactivation with light to initiate intracellular signaling pathways (Airan et al., 2009). One example is opto-α1-adrenergic receptor (AR), which when photostimulated, led to a significant increase in IP_3_ signaling in HEK cells. When this chimeric opsin-receptor was expressed in the nucleus accumbens of mice and photoactivated during a place preference task, mice spent significantly more time in the conditioned area in the subsequent session than control mice without the optical stimulation, indicating that opto-α1-AR can alter cell signaling *in vivo* and affect animal behavior (Airan et al., 2009). This experiment demonstrates the applicability of optogenetics, beyond ionic manipulations, to study effects of metabotropic cell signaling on behavior.

### Single Cell Optogenetics

Neurons in the cortex, even of the same cell type, fire at different times, and this asynchronous firing across space and time is essential for information coding (Goard and Dan, 2009). However, so far, most of the optogenetic applications took reductionist approaches in which all opsin-expressing neurons or axonal projections were activated or inhibited at the same time by uniform light illumination. Because these approaches cannot recapitulate the natural firing patterns of groups of neurons, they limit the understanding of the physiology of neural circuits. Moreover, the dependence of cell-type specific optogenetics on Cre mouse lines limits the freedom of experimental designs. To solve these issues, new techniques which enable selective manipulation of arbitrarily defined neuronal ensembles at a single-cell spatial resolution and at a sub-millisecond temporal resolution are required. In recent years, advances in two directions have been improving fine spatiotemporal optogenetic control: single-cell photostimulation and new opsins of fast kinetics, high conductance, and subcellularly restricted expression. In an early attempt to achieve photostimulation at a single-cell resolution, a small, focal laser spot of blue light was used to raster scan brain tissue in which some neurons expressed ChR2. (Wang et al., 2007). However, this one-photon excitation method suffers from low penetration depth, lack of optical sectioning, and degraded lateral resolution due to light scattering, resulting in unwanted activation of the axons of passage and out of focus somata. To mitigate those issues, the non-linearity of two-photon excitation was used (Rickgauer and Tank, 2009; Prakash et al., 2012). However, traditional two-photon excitation methods are limited by poor temporal precision, as a focused infrared laser must raster scan the surface of opsin-expressing somata to induce enough photocurrent. To surmount the limitation of slow scanning, two parallel illumination methods were invented – computer generated holography (CGH) (Begue et al., 2013; Dal Maschio et al., 2017; Forster et al., 2017; Ronzitti et al., 2017) and generalized phase contrast (GPC) (Papagiakoumou et al., 2010). These techniques rely on phase modulation to make a pattern of infrared illumination precisely matching the three-dimensional profiles of neurons of interest, therefore allowing simultaneous two-photon activation of multiple neuronal targets at once (Gerchberg and Saxton, 1972; Sinclair et al., 2004). When combined with temporal focusing, these parallel illumination methods can reach micrometer resolution even at a depth of hundreds of micrometers in scattering brain tissues. Advances in these state-of-the-art approaches provides a new avenue to systematically map the connectivity of neuronal networks at a cellular resolution.

In addition to the development of single-cell photostimulation, the engineering of new opsins also contributes to single-cell optogenetics. For example, neurons in most of the brain areas are surrounded by numerous neurites of nearby cells, and most methods for expressing opsins will lead to opsin expression in neighboring neurites as well. As a consequence, even with single-cell photostimulation methods mentioned above, the stimulation light will inevitably stimulate dendrites and axons which pass by the targeted neurons. To address this issue, a high-performance somatic opsin, soCoChR, was generated (Shemesh et al., 2017). The expression of the high-photocurrent channelrhodopsin (CoChR) was restricted primarily to the cell bodies of cortical neurons by fusing a short amino-terminal segment of the kainate receptor KA2 subunit to this opsin. With the help of two-photon CGH, soCoChR allows the activation of individual neurons at cellular resolution with sub-millisecond temporal precision. Future work employing both single-cell stimulation and soCoChR will enable more reliable mapping of neuronal connectivity in intact brain circuits.

Moreover, sub-millisecond optogenetics can also be helpful for exploring the connectivity of fast-spiking neurons, such as interneurons (Hajos et al., 2004). Chronos is a fast opsin that can be combined with two-photon parallel illumination to drive spiking up to 100 Hz with sub-millisecond onset precision and cellular resolution. The high spatiotemporal resolution and high rate of optogenetic stimulation exemplified in the above will be essential for faithfully replicating the asynchronous activity in neuronal networks *in vivo*.

### Limitations And Alternative Strategies

Optogenetics is, however, not without limitations. In fact, in some circumstances, it can result in paradoxical and undesirable effects. Inhibitory chloride pumps such as eNpHR can lead to a change in inhibitory signaling that outlasts the photostimulation (Raimondo et al., 2012; Mahn et al., 2016). Long duration photoinhibition through chloride pumps can alter the chloride ion gradient and therefore change GABA_A_ receptor reversal potential, resulting in synaptically evoked spiking following photoinhibition (Raimondo et al., 2012). A similar phenomenon can be seen when using light-driven outward proton pumps such as Arch. Prolonged eNpHR activation overwhelms the neuron’s mechanisms for chloride homeostasis (Raimondo et al., 2012), while prolonged Arch activation can overwhelm the cell’s mechanisms for pH regulation (Mahn et al., 2016). These effects can be mitigated through the use of a pulsing or sinusoidal light stimulus instead of a constant stimulus, by minimizing the intensity and duration of photoinhibition (Raimondo et al., 2012) and by using ramp termination of photostimulation instead of step termination (Mahn et al., 2016).

Another option for photoinhibition is to use ChR2 to activate GABAergic inhibitory neurons. Although this method is more effective than direct photoinhibition by the proton pumps Arch and Jaws (Chuong et al., 2014; Li et al., 2019), it also has its own limitations. For instance, optogenetically activating all GABAergic inhibitory neurons in the cortex will drastically shut down the activity of all principal cells, including various types of cortical projection neurons (e.g., corticocortico, corticothalamic, and corticofugal projections) (Babl et al., 2019). This non-specifical circuit manipulation introduces a confounding factor: we cannot determine if experimental effects result from the direct suppression of the specific pathway of interest or the concurrent suppression of multiple output pathways (Babl et al., 2019). Furthermore, another confounding factor is the rebound activity caused by abruptly terminating the photostimulation of inhibitory neurons. Those synchronized spiking events cross a large number of neurons and can last several seconds, disrupting the functionality of cortical circuits. Therefore, caution should be used when choosing an opsin and when designing a photostimulation method (Liu et al., 2016; Guo et al., 2017).

Moreover, the methods of expression should also be carefully considered. Transgenic lines result in global opsin expression, which leads to at least two complications: first, photostimulation may activate neurons that are located outside of the target region but have axons passing through this region; second, one-photon photostimulation, which has poor spatial resolution, may activate opsin expressing neurons which neighbor the target region. While the use of virus can resolve the issues associated with transgenenic lines by spatially confining opsin expression, some caveats associated with this expression method should also be considered. First, Jackman et al. (2014) reported artificial synaptic depression in hippocampal neurons when ChR2 was expressed using specific AAV virus serotypes, including AAV1, AAV5, and AAV8. But this artifact did not occur when ChR2 was expressed transgenically or with AVV9. Second, some types of viral expression systems are cytotoxic. For instance, the expression of target genes mediated by rabies virus leads to rapid cell death starting 1 week after viral injection (Wickersham et al., 2007), preventing its use for long-term functional experiments. Third, virus-based expression can also be limited by viral tropism in certain brain regions. For example, canine adenovirus and herpes simplex virus 1, two retrograde viruses, labeled largely non-overlapping populations of the basolateral amygdala that project into the prefrontal cortex (Senn et al., 2014). Therefore, one should carefully design experiments, considering the trade-off between transgenic lines and viral methods.

Furthermore, *in vivo* optogenetics is heavily limited by the level of invasiveness. Implementing optogenetics *in vivo* requires either a cranial window for superficial brain areas or an optical fiber implant to deliver light. For deep brain regions, implants also require aspiration of tissue superficial to the target site. Chemogenetics is therefore an appealing alternative as it is effective, minimally invasive, reversible and highly specific. Chemogenetic approaches include Pharmacologically Selective Actuator Modules (PSAMs) and their corresponding Pharmacologically Selective Effector Modules (PSEMs) (Magnus et al., 2011). PSAMs are a toolbox of engineered chimeric ligand-gated ion channels that selectively bind the corresponding, engineered PSEM. For example, combining a pharmacologically selective ion binding domain with the serotonin 3a receptor or with glycine receptors, results in a chimeric channel that selectively binds PSEM and gates depolarizing cation current or hyperpolarizing chloride current respectively. These channels can be expressed *in vivo* and PSEM can be delivered through an intraperitoneal (i.p.) injection (Magnus et al., 2011). Designer Receptors Exclusively Activated by Designer Drugs (DREADDs) are another common chemogenetic toolbox that utilize engineered G-protein coupled receptors to regulate neuronal activity. Two of the most commonly used DREADDs are hM3Dq, an engineered Gq-coupled receptor and hM4di, an engineered Gi-coupled receptor (Armbruster et al., 2007). hM3Dq and hM4Di, which effectively mediate neuronal activation or silencing, respectively, when exposed to their pharmacologically inert ligand, clozapine-N-oxide (CNO) (Armbruster et al., 2007; Alexander et al., 2009; Zhu et al., 2014). For *in vivo* experiments, CNO can be delivered via an i.p. injection to perturb the activity of all neurons which express DREADDs. Alternatively, when DREADDs are expressed in specific populations of projection neurons and CNO is be locally infused in a post-synaptic region, projection-specific activation (Vazey and Aston-Jones, 2014) or silencing (Mahler et al., 2014; Stachniak et al., 2014; Zhu et al., 2016) of axon terminals can be achieved. However, it is worth noting that CNO has dose-dependent side effects: excessive CNO is converted to clozapine and could alter animal behavior independent of DREADDs (Manvich et al., 2018). These side effects can be reduced by using an appropriate dosage of CNO. Overall, chemogenetics offers many of the same advantages as optogenetics but has the additional advantage of being minimally-invasive, making it an ideal technique for targeting deep brain structures. When combined with an intersectional approach, chemogenetics can also be used for cell-type and projection-specific dissection. Chemogenetics however, lacks the fast kinetics of optogenetics, therefore limiting its applications in reversible circuit manipulation.

Other less prevalent solutions to the invasiveness of optogenetics also exist. Transcranial near-infrared optogenetics utilizes synthesized Lanthanide-doped upconversion nanoparticles (UCNPs), which convert near infrared photons into high energy visible light sufficient to activate opsins (Chen et al., 2018). For example, the authors expressed ChR2 in the VTA, and injected UCNPs into the VTA. Afterward, they delivered near infrared light outside of the skull, which was sufficient to evoke firing in ChR2 expressing neurons in the VTA (Chen et al., 2018). Additionally, magnetic manipulation of neural activity, dubbed Magneto2.0 (Wheeler et al., 2016), also offers a non-invasive solution; however, it remains unclear whether this approach can reliably manipulate neuronal circuits in various brain structures, as conventional optogenetic methods have (Meister, 2016; Kole et al., 2019; Wang et al., 2019; Xu et al., 2019). In conclusion, experimentalists now have numerous techniques at their finger tips for flexible manipulations of neuron activity both *in vitro* and *in vivo*. Among these techniques optogenetics provides numerous powerful advantages for modern circuit dissection.

## Author Contributions

All authors contributed to writing the review.

## Conflict of Interest

The authors declare that the research was conducted in the absence of any commercial or financial relationships that could be construed as a potential conflict of interest.
